# NON-ADHERENCE TO FOLLOW-UP CARE IN PERSONS WITH SPINAL CORD INJURY WITHIN 10 YEARS AFTER INITIAL REHABILITATION

**DOI:** 10.2340/jrm.v56.41083

**Published:** 2024-08-26

**Authors:** Inge E. ERIKS-HOOGLAND, Lorena MÜLLER, Benjamin D. N. HIRSCH, Lea STUDER, Armin GEMPERLI, Collene E. ANDERSON

**Affiliations:** 1 Swiss Paraplegic Centre, Nottwil; 2 Swiss Paraplegic Research, Nottwil; 3 Department of Health Sciences and Medicine, University of Lucerne, Lucerne; 4 Center of Primary and Community Care, University of Lucerne, Lucerne; 5 Department of Neuro-Urology, Balgrist University Hospital, University of Zurich, Zurich, Switzerland

**Keywords:** follow-up care, secondary health conditions, secondary prevention, spinal cord injury

## Abstract

**Objective:**

This study aimed to describe the temporal dynamics of and risk factors for non-adherence to outpatient follow-up care in the first 10 years after spinal cord injury.

**Design:**

Retrospective single-centre cohort study using data from medical records and municipal resident registers.

**Subjects/Patients:**

Patients admitted to a specialized spinal cord injury centre in Switzerland discharged between 1 January 2010 and 31 December 2012 (*n* = 225). Time-to-event analysis was used to investigate the timing of the first non-adherence event, its association with spinal cord injury, and sociodemographic characteristics.

**Results:**

36% of patients were adherent to annual follow-up appointments; 2% formally transferred to another SCI centre; 44% were non-adherent for general reasons (patient’s will to discontinue care [12%] or unknown reasons [32%]); and 18% were non-adherent due to death. Risk factors for non-adherence included older age, lack of long-term partner, and more than 2 h of travel time to the clinic. In the youngest age group (18–30 years), 55% were non-adherent after 10 years.

**Conclusion:**

A relevant proportion of individuals with spinal cord injury were lost to annual follow-up care. A holistic approach to patient engagement integrating solutions such as telemedicine and involvement of support networks could reduce the risk of non-adherence.

## LAY ABSTRACT

Regular follow-up appointments are crucial for individuals with spinal cord injuries for monitoring health and preventing complications. However, many individuals fail to adhere to recommended care. This study examined non-adherence to follow-up appointments within 10 years post-injury and identified risk factors. Analysing data from 225 people admitted for spinal cord injury rehabilitation to a Swiss centre between 2010 and 2012, researchers found that only 36% consistently attended annual follow-ups over a decade; 32% stopped attending for unknown reasons, 12% discontinued specialized care, 2% transferred to another clinic, and 18% passed away. Factors associated with loss to follow-up included older age, car travel time exceeding 2 h, and lack of a long-term partner. The study underscores the importance of understanding barriers to receiving recommended care for persons with spinal cord injury. Identifying high-risk groups is crucial for developing strategies to improve adherence and enhance long-term outcomes in this population.

Spinal cord injury (SCI) is a life-altering event that results in significant physical, psychological, and social challenges for affected individuals. Comprehensive and continuous healthcare management across the lifespan is crucial to optimize health and rehabilitation outcomes and maximize the quality of life for those with SCI, as recommended by the clinical practice guideline on follow-up care for persons with SCI (1–4). Non-adherence to follow-up care is a known problem in chronic disease (5). Attrition in follow-up care, or non-adherence to follow-up care, refers to individuals disengaging or failing to attend scheduled medical appointments, leading to interrupted or incomplete healthcare management. Consequences can range from suboptimal recovery to exacerbation of secondary health conditions (SHCs) including multiple potentially modifiable conditions, such as pressure injuries (6), urinary tract infections (UTI) (7), autonomic dysreflexia (7), and cardiovascular and metabolic disease (8). Finally, mortality among persons with SCI not attending annual check-ups was found to be nearly fourfold higher than those attending regular check-ups (9).

Therefore, annual check-up appointments at specialized SCI centres are recommended. In Switzerland, Germany, and Austria, interdisciplinary follow-up care appointments after primary rehabilitation are planned in specialized SCI clinics at 3, 6, and 12 months after discharge, continuing annually (1). Check-ups include, based on the International Classification of Functioning and Health, comprehensive evaluation of neurological status, general health status, SCI-related complications, and rehabilitation status (specified for age, lesion level, sex, and completeness of SCI). This includes an anamnesis, physical examination, and additional tests as recommended by the guideline. Despite advancements in SCI care, rehabilitation, and knowledge of the importance of attending follow-up care appointments, the issue of “non-adherence to follow-up care” remains a persistent concern. Female gender, incomplete SCI, and long travel time are risk factors for non-adherence (10–12), but because these findings were assessed by a cross-sectional survey in the community setting, they have limited persuasiveness due to non-response bias and lack of longitudinal assessment. Understanding the determinants of non-adherence is crucial in mitigating its impact on persons with SCI. Identifying and addressing these determinants can enable healthcare providers to develop targeted interventions and policies aimed at reducing attrition rates and improving patient engagement throughout the continuum of care.

Our study aims to describe the occurrence of non-adherence to follow-up care after primary rehabilitation in individuals with SCI. We will explore the timing of non-adherence in relation to various determinants (including but not limited to injury characteristics, sociodemographic factors, healthcare system-related challenges, support networks, and geographical limitations) that potentially contribute to non-adherence over 10 years after primary inpatient rehabilitation. Ultimately, our goal is to use a learning health system approach, to provide a valuable resource for clinicians, researchers, and policymakers to develop evidence-based strategies for enhancing continuity of care and optimizing outcomes in this vulnerable population.

## METHODS

### Study design

This was a retrospective single-centre cohort study in persons who underwent specialized rehabilitation for a newly acquired SCI with at least a 10-year follow-up after discharge.

### Setting

This study was conducted within a specialized SCI hospital and rehabilitation centre in Switzerland. The study site represents the largest (> 200 beds) of 4 SCI specialized clinics in Switzerland. Costs for follow-up care are fully covered by health insurance or accident insurance (including all travel expenses).

### Participants

All adults with SCI admitted for initial rehabilitation and discharged between 1 January 2010 and 31 December 2012 were included. Individuals who refused consent to retrospective use of medical data, had diseases other than SCI (e.g., Guillain-Barré syndrome, critical illness polyneuropathy, stroke, multiple sclerosis, functional neurological disorder), or were under the age of 18 years at date of hospitalization were excluded from our study. Persons who were not Swiss residents, or who moved abroad directly after rehabilitation, were also excluded.

### Data sources/measurement

All data used in this study originate from medical records, a hospital outpatient appointment planning tool (Polypoint), and the municipal resident registers. Adherence status was assessed based on information from the clinical record and the outpatient planning tool extracted between June 2022 and February 2023, making it possible for all included patients to have at least 10 years of follow-up time. Attempts were made to contact patients who were non-adherent or their relatives/responsible parties at the time of data extraction by phone between September 2022 and October 2022. In cases where patients were successfully reached, reasons for non-adherence (bad health condition, transport problem, follow-up care in another institution, no need for or interest in follow-up care) and their place of residence were requested. If patients could not be contacted directly, available relatives or responsible parties (e.g., nursing staff responsible in a nursing home) were asked about the patient’s vital status (alive or dead), including the precise date of death in the event they had passed away. Data were transferred to a SecuTrial database by 2 research assistants (BH, LS).

### Main outcome measures

*Non-adherence.* Non-adherence was defined as not attending annual interdisciplinary follow-up appointments for three consecutive years (1095 days). This time frame was chosen because current practice in our setting recommends annual follow-up appointments but due to variation in the waiting list length, appointments might not be scheduled exactly after 1 year. Also, based on clinical expertise, missing one or even two appointments were not directly considered as loss to follow-up.

*Risk factors for non-adherence.* Potential risk factors for non-adherence were selected based on previous literature (10, 11, 13) and included:

- demographic variables: age at injury, sex, travel time to the clinic by car (min), partnership status (married or partnership/not married or no partnership), language region (Italian speaking, French speaking, German speaking);
- lesion characteristics: aetiology of injury (traumatic/non-traumatic), injury severity (paraplegia/tetraplegia – complete/incomplete);
- other variables were: functional independence – Spinal Cord Independence Measure (SCIM) III Score (0–100) and presence of comorbidities derived from the Charlson Comorbidity Index (CCI) (14). As the information needed to calculate the CCI was not fully available in the clinical record, based on expert input the following comorbidity groups were utilized in the analyses: cancer, cardiological, mental, neurological, pulmonary.

*Ethical approval.* The study was formally approved and performed in adherence to the guidelines of the regional medical ethics committee of northwest and central Switzerland (BASEC-Nr. 2022-00435). All the participants included in this study were informed about the possible future use of their medical data and had the option to opt out.

### Modelling considerations

Time-to-event analyses were used to describe the first occurrence of non-adherence, under the assumption that the patients are missing out on relevant medical care and the tendency towards non-adherence is a stable trait. Non-adherence due to death was identified as a competing risk of non-adherence for all other causes, as death precludes other forms of non-adherence, and has different implications for clinical planning and preventative interventions. Discharge date is taken as the starting point for time at risk, as it is the reference date for scheduling follow-up assessments in the local clinic (clinical guidelines [1]). Event times for all outcomes were designated as the last visit where the patient was present at the outpatient clinic or from the date of discharge if patients did not return. Exact death dates were unknown in some cases and might reflect the date that the clinic was informed of the death in others. Patients in the adherent group were right-censored at the time of their last outpatient visit, as were patients who formally transferred to another SCI clinic.

### Statistical methods

The population was stratified into 3 adherence status groups (adherent, non-adherent for general reasons, and non-adherent due to death) for descriptive statistics. Continuous variables were described using medians and quartiles as variables were not normally distributed (confirmed by visual inspection and normality testing). Differences between medians are displayed with bootstrapped 95% confidence intervals (1,000 iterations). Confidence intervals for differences in proportions are based on a two-sample test of proportions using Bonferroni-corrections for categorical variables with multiple levels.

To account for the competing risk events appropriately, cumulative incidence functions (CIFs) are used to visualize univariable time-to-event results. Semiparametric multivariable Cox regressions were used to calculate cause-specific hazard ratios for risk factors for non-adherence: (*i*) non-adherence due to causes other than death (general non-adherence) and (*ii*) non-adherence due to death. In both cases of cause-specific non-adherence, the competing risk outcome was treated as right censoring. The resulting cause-specific hazard ratios should be interpreted as describing the association between risk factors and the rate at which the respective events occur over time (15). A semiparametric multivariable competing risks regression based on the Fine and Gray method (16) was used to estimate subdistribution hazard ratios for risk factors for the first event of non-adherence, accounting for death. The subhazard ratios should be interpreted as indicating which covariates are associated with the cumulative incidence function, or probability that non-adherence will occur over time, accounting for death (15). The proportional hazards assumption was assessed using Schoenfeld residuals (global and variable-level tests did not indicate violations), and crude cumulative incidence functions (possible proportional hazard violations in age and SCIM score). Continuous specification of age (17) and SCIM score III (divided into tertiles) were tested; categorical specification is displayed for clearer communication, as categorical and continuous models produced comparable results. Global statistical significance was evaluated using Wald tests in cases where categorical variables had more than 2 levels. The primary analyses use multiple imputation (MI) with chained equations to account for missing values (18, 19); complete-case analyses are included in the supplementary material (Tables SI and SII). Analyses were performed in Stata Statistical Software, Release 18, 2023 (StataCorp LLC, College Station, TX, USA). Ado packages applied for the competing risks survival analysis included: stcompet for crude cumulative incidence functions (20), merlin (21), and predictms (22) for adjusted cumulative incidence functions (simulation estimator).

## RESULTS

### Descriptive data (including participant information)

**Fig. 1:**
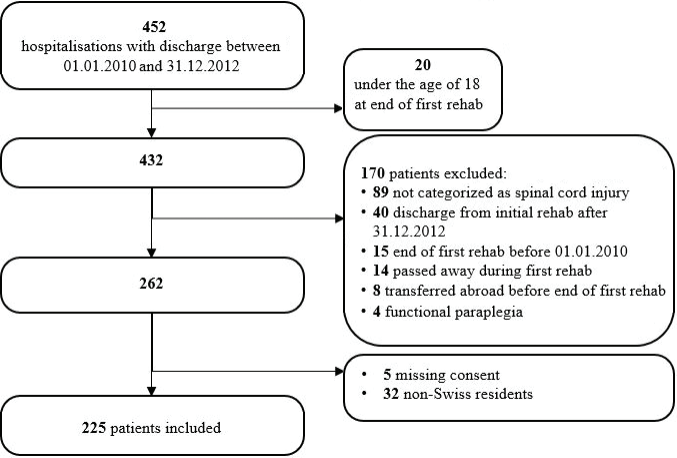
Participant inclusion flowchart.

A total of 452 individuals were admitted in the defined period to the study centre, 168 of whom were not eligible due to age, medical diagnosis, or discharge time frame. Twenty-two persons were excluded because of transfer or death before the end of rehabilitation. A further 32 were excluded because they lived outside Switzerland after discharge, and 5 were excluded due to a lack of consent (Fig. 1).

Of the 225 patients included, 153 (68%) were male with a median (interquartile range [IQR]) population age of 52 years (33–66) at the time of SCI. In 76% aetiology of SCI was traumatic, 15% of the population was classified as C1–4, AIS A–C lesion, 11% C5–8, AIS A–C lesion, 35% T1–S1 AIS A–C lesion and 39% of the population were classified AIS D. Length of stay was a median IQR of 173 days (134–235), median (IQR) SCIM score at discharge was 68 (38–81), and 176 patients (78%) were discharged to their own home. Table I gives a detailed overview of the study population overall, and stratified on adherence status.

**Table I:** Characteristics of the study population at the time of discharge from rehabilitation

Characteristic	Overall population *n* = 225Median (IQR)	Adherent *n* = 85Median (IQR)	Non-adherent, general *n* = 99Median (IQR)	Difference non-adherent–adherentMedians (95% CI)	Non-adherent due to death *n* = 41Median (IQR)	Difference death– adherentMedians (95% CI)
Age at SCI	52 (33–66)	44 (33–55)	50 (29–66)	6 (–5, 17)	68 (61–77)	24 (17, 31)
Length of stay (days)	178 (134–235)	184 (163–250)	162 (120–188)	–22 (–52, 8)	192 (148–243)	8 (–31, 47)
SCIM III score, total	68 (38–81)	70 (53–83)	71 (47–86)	1 (–5, 7)	32 (21–59)	–38 (–48, –28)
Categorical variables	N (%)	N (%)	N (%)	Difference in proportions (95% CI)	N (%)	Difference in proportions (95% CI)
Age at Spinal cord injury						
18–30 years	52 (23)	20 (24)	29 (29)	0.06 (–0.11, 0.22)	3 (7)	–0.16 (–0.32, 0.00)
31–45 years	44 (20)	27 (32)	16 (16)	–0.16 (–0.32, 0.01)	1 (2)	–0.29 (–0.44, –0.15)
46–60 years	47 (21)	23 (27)	18 (18)	–0.09 (–0.25, 0.07)	6 (15)	–0.12 (–0.31, 0.06)
61–75 years	58 (26)	15 (18)	25 (25)	0.08 (–0.08, 0.23)	18 (44)	0.26 (0.04, 0.49)
≥76 years	24 (11)	0 (0)	11 (11)	0.11 (0.03, 0.19)	13 (32)	0.32 (0.13, 0.50)
Sex						
Male	153 (68)	60 (71)	61 (62)	–0.09 (–0.25, 0.07)	32 (78)	0.07 (–0.11, 0.26)
Female	72 (32)	25 (29)	38 (38)	0.09 (–0.07, 0.25)	9 (22)	–0.07 (–0.26, 0.11)
Language region						
German	176 (78)	68 (80)	74 (75)	–0.05 (–0.20, 0.09)	34 (83)	0.03 (–0.15, 0.20)
French	40 (18)	15 (18)	18 (18)	0.01 (–0.13, 0.14)	7 (17)	–0.01 (–0.18, 0.17)
Italian	9 (4)	2 (2)	7 (7)	0.05 (–0.03, 0.12)	0 (0)	–0.02 (–0.06, 0.02)
Car travel time						
0–1 h	101 (45)	40 (47)	42 (42)	–0.05 (–0.23, 0.14)	19 (46)	–0.01 (–0.24, 0.23)
1–2 h	86 (38)	35 (41)	35 (35)	–0.06 (–0.24, 0.12)	16 (39)	–0.02 (–0.25, 0.21)
2–3 h	35 (16)	9 (11)	20 (20)	0.10 (–0.03, 0.23)	6 (15)	0.04 (–0.12, 0.20)
> 3 h	3 (1)	1 (1)	2 (2)	0.01 (–0.04, 0.05)	0 (0)	–0.01 (–0.04, 0.02)
Living situation after discharge						
Own home – with family	138 (61)	60 (71)	58 (59)	–0.12 (–0.30, 0.06)	20 (49)	–0.22 (–0.46, 0.02)
Own home – alone	38 (17)	16 (19)	19 (19)	0.00 (–0.15, 0.15)	3 (7)	–0.12 (–0.27, 0.04)
Assisted living	6 (3)	3 (4)	3 (3)	0.00 (–0.07, 0.06)	0 (0)	–0.04 (–0.09, 0.02)
Institution	41 (18)	6 (7)	17 (17)	0.10 (–0.02, 0.22)	18 (44)	0.37 (0.16, 0.58)
Unknown	2 (1)	0 (0)	2 (2)	0.02 (–0.02, 0.06)	0 (0)	0.00 (0.00, 0.00)
Civil status						
Married	126 (56)	52 (61)	47 (47)	–0.14 (–0.32, 0.05)	27 (66)	0.05 (–0.18, 0.27)
Not married	74 (33)	30 (35)	38 (38)	0.03 (–0.15, 0.21)	6 (15)	–0.21 (–0.40, –0.02)
Other	13 (6)	3 (4)	8 (8)	0.05 (–0.04, 0.13)	2 (5)	0.01 (–0.08, 0.11)
Unknown	12 (5)	0 (0)	6 (6)	0.06 (0.00, 0.12)	6 (15)	0.15 (0.01, 0.28)
Employment before Spinal cord injury						
Paid work	125 (56)	63 (74)	52 (53)	–0.22 (–0.38, –0.05)	10 (24)	–0.50 (–0.69, –0.30)
No paid work	95 (42)	21 (25)	43 (43)	0.19 (0.02, 0.35)	31 (76)	0.51 (0.31, 0.70)
Unknown	5 (2)	1 (1)	4 (4)	0.03 (–0.03, 0.08)	0 (0)	–0.01 (–0.04, 0.02)
Length of stay, categorized						
0–3 months	23 (10)	7 (8)	16 (16)	0.08 (–0.04, 0.20)	0 (0)	–0.08 (–0.16, –0.01)
3–6 months	109 (48)	35 (41)	56 (57)	0.15 (–0.03, 0.34)	18 (44)	0.03 (–0.21, 0.26)
6–9 months	62 (28)	30 (35)	17 (17)	–0.18 (–0.34, –0.02)	15 (37)	0.01 (–0.22, 0.24)
> 9 months	31 (14)	13 (15)	10 (10)	–0.05 (–0.18, 0.07)	8 (20)	0.04 (–0.14, 0.22)
SCIM III score, categorized						
0–25	35 (16)	10 (12)	11 (11)	–0.01 (–0.12, 0.11)	14 (34)	0.22 (0.02, 0.43)
26–50	42 (19)	11 (13)	17 (17)	0.04 (–0.09, 0.17)	14 (34)	0.21 (0.01, 0.42)
51–75	71 (32)	32 (38)	30 (30)	–0.07 (–0.25, 0.10)	9 (22)	–0.16 (–0.37, 0.05)
76–100	77 (34)	32 (38)	41 (41)	0.04 (–0.14, 0.22)	4 (10)	–0.28 (–0.45, –0.10)
Lesion aetiology						
TSCI	170 (76)	71 (84)	74 (75)	–0.09 (–0.22, 0.05)	25 (61)	–0.23 (–0.42, –0.03)
NTSCI	55 (24)	14 (16)	25 (25)	0.09 (–0.05, 0.22)	16 (39)	0.23 (0.03, 0.42)
SCI severity						
C1–C4 AIS A,B,C	34 (15)	11 (13)	12 (12)	–0.01 (–0.13, 0.11)	11 (27)	0.14 (–0.06, 0.33)
C5–C8 AIS A,B,C	24 (11)	11 (13)	6 (6)	–0.07 (–0.18, 0.04)	7 (17)	0.04 (–0.13, 0.21)
T1–S1 AIS A,B,C	79 (35)	32 (38)	34 (34)	–0.03 (–0.21, 0.14)	13 (32)	–0.06 (–0.28, 0.16)
All AIS D	88 (39)	31 (36)	47 (47)	0.11 (–0.07, 0.29)	10 (24)	–0.12 (–0.33, 0.09)
Ventilator						
None	187 (83)	77 (91)	82 (83)	–0.08 (–0.20, 0.05)	28 (68)	–0.22 (–0.43, –0.02)
CPAP/BiPAP	29 (13)	7 (8)	14 (14)	0.06 (–0.06, 0.18)	8 (20)	0.11 (–0.06, 0.29)
Yes, < 24 h per day	3 (1)	0 (0)	0 (0)	0.00 (0.00, 0.00)	3 (7)	0.07 (–0.03, 0.18)
Yes, 24 h per day	4 (2)	0 (0)	2 (2)	0.02 (–0.02, 0.06)	2 (5)	0.05 (–0.04, 0.14)
Yes, unknown hours per day	2 (1)	1 (1)	1 (1)	0.00 (–0.04, 0.04)	0 (0)	–0.01 (–0.04, 0.02)
Comorbidities present						
Cancer	28 (12)	5 (6)	8 (8)	0.02 (–0.05, 0.10)	15 (37)	0.31 (0.15, 0.46)
Cardiovascular	150 (67)	59 (69)	56 (57)	–0.13 (–0.27, 0.01)	35 (85)	0.16 (0.01, 0.31)
Genitourinary	212 (94)	83 (98)	90 (91)	–0.07 (–0.13, 0.00)	39 (95)	–0.03 (–0.10, 0.05)
Mental	76 (34)	27 (32)	32 (32)	0.01 (–0.13, 0.14)	17 (41)	0.10 (–0.08, 0.28)
Neurological	159 (71)	62 (73)	64 (65)	–0.08 (–0.22, 0.05)	33 (80)	0.08 (–0.08, 0.23)
Pressure injury	58 (26)	23 (27)	18 (18)	–0.09 (–0.21, 0.03)	17 (41)	0.14 (–0.03, 0.32)
Pulmonary	124 (55)	38 (45)	55 (56)	0.11 (–0.04, 0.25)	31 (76)	0.31 (0.14, 0.48)

Difference in medians is calculated with bootstrapped 95% confidence intervals, difference in proportions is based on a 2-sample test of proportions, with Bonferroni-corrected confidence intervals for categorical variables with multiple levels.

IQR: interquartile range; SCIM: Spinal Cord Independence Measure; CPAP: continuous positive airway pressure; BiPAP: bi-level positive airway pressure.

### Non-adherence

*Non-adherence 10 years after discharge.* Ten years after initial rehabilitation, 36% of the study population (*n* = 81) were still adhering to the recommended annual follow-up appointments. An additional 4 patients (2%) who formally transferred to other SCI clinics for follow-up care were categorized as part of the adherent group. Of the 62% (*n* = 140) that were defined as non-adherent, 26 individuals (12%) chose not to return for follow-up visits, 22 of whom reasoned that they had such a positive recovery they felt no specialized SCI follow-up was needed, with a subgroup (*n* = 6) indicating that they were visiting their general practitioner for continued medical care. Other reasons for not returning for outpatient follow-up were too long a travel time (*n* = 2), and a poor health condition (*n* = 2). In *n* = 73 patients (32%) we have no information on reasons for not attending follow-up care appointments. Finally, 41 persons (18% of the overall population, 29% of the non-adherent population) were categorized as non-adherent to follow-up visits due to death within the study follow-up period.

As sensitivity analysis, if a definition of non-adherence is taken where inpatient stays are also counted towards adherence time, *n* = 16 patients considered non-adherent for general reasons in the primary analysis change status (12 to adherent, 4 to non-adherent due to death). The median (IQR) age of these patients was 52 (27–65) and *n* = 8 were male. The group consisted of *n* = 7 C1–C8 AIS A–C, *n* = 8 T1–S1 AIS A–C, and *n* = 1 AIS D patient, with a median (IQR) SCIM score of 66 (34–71).

**Fig. 2:**
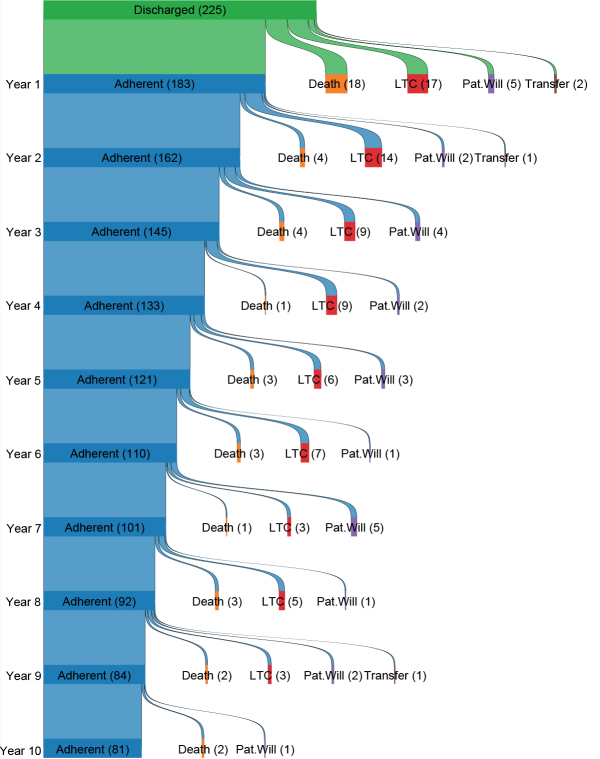
Sankey diagram illustrating adherence status during the first 10 years after SCI. Patients are defined as “adherent” for a given year if the final outpatient clinic visit was up to 6 months before the year mark, e.g., someone with a final visit 1.5 years after discharge (548 days) would be considered adherent for years 1 and 2, but not for year 3. Year 1 is an exception; patients are marked as non-adherent at year 1 if the final clinic visit was <274 days after discharge. LTC=lost to clinic. Pat. will = patient’s will. Transfer = formal transfer to another SCI clinic.

*Pattern of non-adherence.* The percentage of patients with a first occurrence of non-adherence to follow-up care was highest in the first year after discharge from rehabilitation, with 18% of the patients already failing to return for the 1-year appointment. First occurrence of non-adherence then dropped to 11% for year 1–2, and 10% for year 2–3, then stabilized between 8% and 9% for years 3–8. Almost half of the cases that were non-adherent due to death (18/41, 44%) during the 10-year study follow-up period already did not return for the 1-year follow-up appointment. Dynamics of the first non-adherence event are shown in detail in Fig. 2.

Some of those classified as non-adherent within the definition of this study later returned to the clinic or passed away. Thirty-four (34/81, 42%) patients who were classified as non-adherent eventually returned to the outpatient clinic. They became non-adherent a median (IQR) of 3.1 (1.7–5.4) years after SCI and returned to the outpatient clinic a median (IQR) of 8.0 (6.0–9.5) years after SCI. This population had a median (IQR) age of 44 years (27–59), with *n* = 18 males (53%). Median (IQR) SCIM score was 71 (47–88), and the population consisted of *n* = 7 C1–C8 AIS A–C, *n* = 12 T1–S1 AIS A–C, and *n* = 15 AIS D patients. Additionally, 16/81 (20%) in the general non-adherence group died after having not visited the clinic for at least 3 years. These patients passed away a median (IQR) of 7.5 (4.1–9.2) years after SCI, had a median (IQR) age of 72 years at SCI (61–83), and 12 were male. SCIM scores in this group tended to be lower than those of the overall population (46 [17–68)]), with SCI severity distribution as follows: *n* = 7 C1–C8 AIS A–C; *n* = 4 T1–S1 AIS A–C; and *n* = 5 with AIS D SCI.

**Fig. 3:**
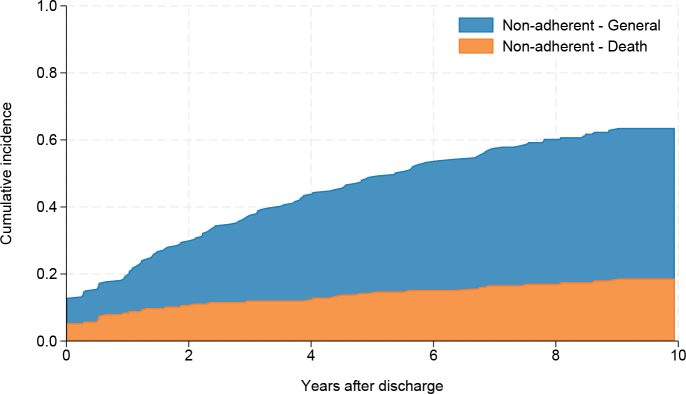
Cumulative incidence function (CIF) for first occurrence of non-adherence during the 10 years after discharge from SCI rehabilitation. General non-adherence (due to patient will, health state, or reasons unknown) is shown in blue, non-adherence due to death is in orange. End of observation time for all groups is the final outpatient follow-up visit.

The adherent group contributed 798 years of time at risk, with a median (IQR) follow-up time in the outpatient clinic of 9.8 years (8.9–10.4, max 12.1). The general non-adherence group contributed 291 years of time at risk, with a median (IQR) follow-up time of 2.4 years (1.0–4.8, max 8.9). The group that was non-adherent due to death contributed 104 years of time at risk with a median (IQR) follow-up time in the clinic of 1.2 years (0.0–4.5, max 9.0). End of observation time for all groups was defined as the final follow-up visit in the outpatient clinic or discharge from initial rehabilitation if patients failed to return for any outpatient visits. The cumulative incidence function for the first occurrence of general non-adherence and non-adherence due to death is displayed in Fig. 3.

### Risk factors for non-adherence

**Fig. 4:**
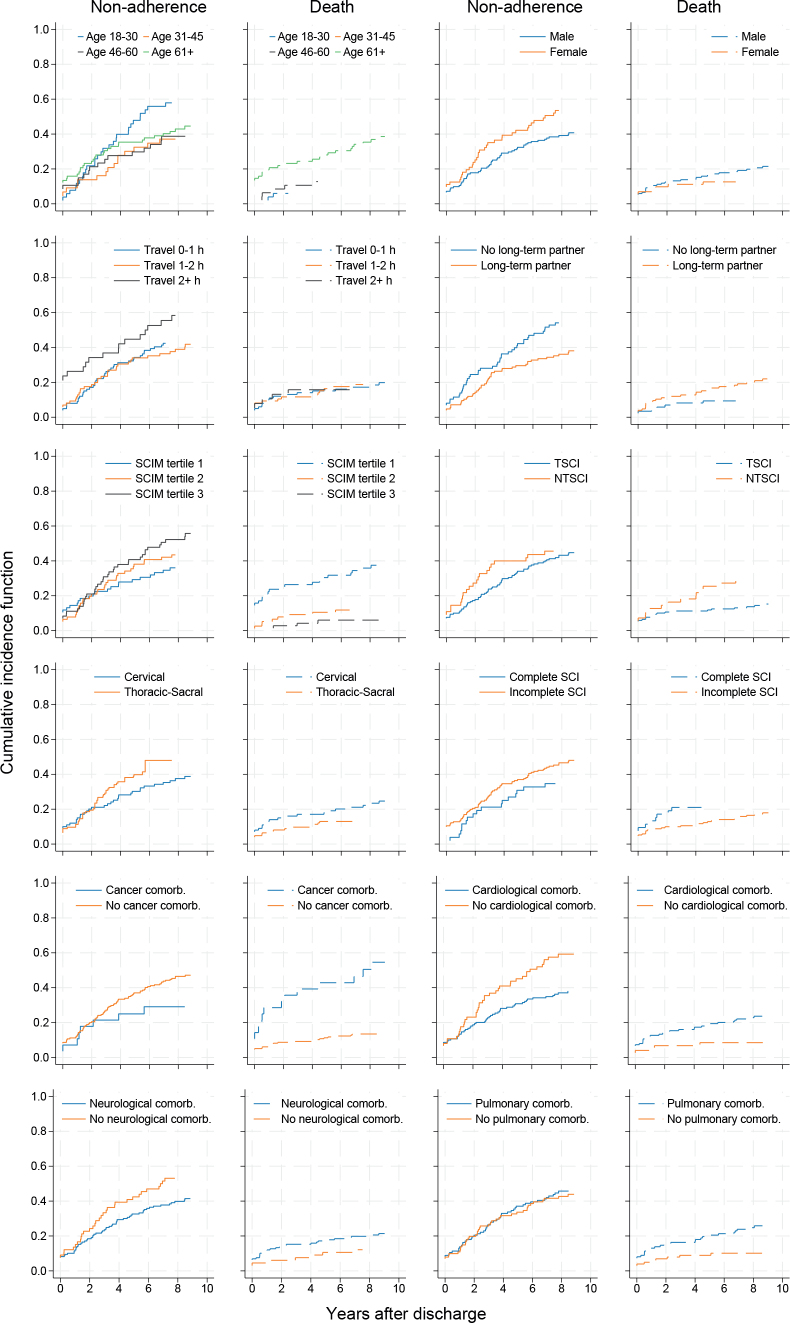
Cumulative incidence functions of general non-adherence and non-adherence due to death stratified for sociodemographic, SCI, and comorbidity risk factors (status at discharge). Comorb.: comorbidity.

Cumulative incidence functions for general non-adherence and non-adherence due to death are shown stratified according to risk factor groups in Fig. 4. Cause-specific regression analyses for general non-adherence identified risk factors associated with the rate of non-adherence in patients who were still alive (Table II, Table SI). Age was a relevant risk factor (global *p* = 0.050); hazard of non-adherence was estimated to be higher in the 61+ (aHR 2.71 [95% CI: 1.24–5.93]) age group than in the 31–45 age group. Comorbidities had varying associations with non-adherence: presence of a pulmonary comorbidity was a risk factor for non-adherence in multivariable analysis (aHR 1.74 [95% CI: 1.09–2.79]). Conversely, persons with a cardiological comorbidity were more likely to return for outpatient check-ups (aHR 0.51 [95% CI: 0.32–0.81]) than those without a cardiological comorbidity, and presence of cancer, mental, and neurological diagnoses were not associated with the risk of non-adherence. Persons who were not in a long-term partnership were also less likely to adhere to outpatient check-ups than persons in a partnership (aHR 1.87 [95% CI: 1.10–3.18]). Travel time also had a tentative association with non-adherence (global *p* = 0.06): the group that had to travel more than 2 h was estimated to be at a higher risk than the group that had travelled less than 1 h (aHR 1.72 [95% CI: 1.03–2.87]).

**Table II:** Risk factors for non-adherence to outpatient follow-up

Risk factor	Univariable HR (95% CI)	*p*-value	Adjusted HR (95% CI)	*p*-value	Univariable sub-HR (95% CI)	*p*-value	Adjusted sub-HR (95% CI)	*p*-value
Age at spinal cord injury		0.07		0.050		0.16		0.12
18–30 years	1.91 (1.04–3.49)		1.72 (0.92–3.20)		1.80 (1.00–3.25)		1.58 (0.86–2.88)	
31–45 years	Reference		Reference		Reference		Reference	
46–60 years	1.16 (0.59–2.28)		1.49 (0.68–3.26)		1.06 (0.55–2.07)		1.48 (0.68–3.22)	
≥61 years	1.85 (1.02–3.33)		2.71 (1.24–5.93)		1.30 (0.73–2.34)		2.34 (1.11–4.94)	
Sex		0.09		0.26		0.06		0.14
Male	Reference		Reference		Reference		Reference	
Female	1.42 (0.95–2.12)		1.28 (0.83–1.98)		1.46 (0.98–2.19)		1.39 (0.90–2.14)	
Car travel time		0.11		0.062		0.14		0.13
0–1 h	Reference		Reference		Reference		Reference	
1–2 h	0.95 (0.61–1.48)		0.89 (0.56–1.42)		0.96 (0.62–1.50)		0.86 (0.54–1.37)	
2+ h	1.61 (0.97–2.68)		1.72 (1.03–2.87)		1.57 (0.94–2.63)		1.53 (0.92–2.55)	
Partnership status on discharge		0.027		0.020		0.011		0.018
No long-term partner	1.56 (1.05–2.33)		1.87 (1.10–3.18)		1.69 (1.13–2.54)		1.94 (1.12–3.35)	
Long-term partner	Reference		Reference		Reference		Reference	
SCIM III score		0.60		0.81		0.12		0.32
Tertile 1 (0–48)	0.84 (0.52–1.36)		0.82 (0.42–1.61)		0.60 (0.37–0.98)		0.60 (0.31–1.16)	
Tertile 2 (49–76)	0.80 (0.51–1.27)		0.87 (0.52–1.46)		0.75 (0.48–1.18)		0.85 (0.51–1.42)	
Tertile 3 (77–100)	Reference		Reference		Reference		Reference	
Lesion aetiology		0.28		0.59		0.64		0.77
Non-traumatic spinal cord injury	1.29 (0.81–2.05)		1.17 (0.66–2.09)		1.12 (0.70–1.78)		1.09 (0.62–1.92)	
Traumatic spinal cord injury	Reference		Reference		Reference		Reference	
Neurological level		0.22		0.94		0.12		0.88
Cervical	0.78 (0.52–1.16)		0.98 (0.56–1.72)		0.72 (0.48–1.08)		1.04 (0.61–1.79)	
Thoracic-sacral	Reference		Reference		Reference		Reference	
Completeness		0.19		0.13		0.11		0.059
Complete (AIS A)	0.71 (0.43–1.18)		0.61 (0.33–1.15)		0.66 (0.40–1.10)		0.55 (0.30–1.02)	
Incomplete (AIS B–E)	Reference		Reference		Reference		Reference	
Comorbidity, cancer		0.45		0.61		0.10		0.21
No	Reference		Reference		Reference		Reference	
Yes	0.76 (0.37–1.55)		0.81 (0.37–1.80)		0.54 (0.26–1.13)		0.61 (0.28–1.33)	
Comorbidity, cardiological		0.023		< 0.01		< 0.01		< 0.01
No	Reference		Reference		Reference		Reference	
Yes	0.64 (0.43–0.94)		0.51 (0.32–0.81)		0.57 (0.38–0.84)		0.54 (0.34–0.86)	
Comorbidity, mental		0.96		0.82		0.76		0.74
No	Reference		Reference		Reference		Reference	
Yes	1.01 (0.66–1.54)		1.06 (0.66–1.70)		0.94 (0.61–1.43)		0.92 (0.57–1.48)	
Comorbidity, neurological		0.22		0.81		0.09		> 0.99
No	Reference		Reference		Reference		Reference	
Yes	0.78 (0.52–1.17)		1.06 (0.66–1.70)		0.71 (0.47–1.06)		1.00 (0.63–1.59)	
Comorbidity, pulmonary		0.32		0.021		0.79		0.026
No	Reference		Reference		Reference		Reference	
Yes	1.22 (0.82–1.81)		1.74 (1.09–2.79)		1.06 (0.71–1.56)		1.70 (1.07–2.71)	

Unadjusted and adjusted hazard ratios from a cause-specific Cox regression with death treated as non-informative censoring, as well as unadjusted and adjusted subhazard ratios from a competing risk regression treating death as a competing event. All listed covariates were included in the adjusted analyses; multiple imputation was used to account for missing data.

HR: hazard ratio; SCIM: Spinal Cord Independence Measure.

Cause-specific regression analyses for non-adherence due to death, among patients who were adherent to treatment (i.e., not lost to clinic), identified age (aHR 61+ vs 31–45 18.47 [95% CI: 1.38–246.39]) as being associated with the rate of non-adherence due to death. Sex (aHR female 0.35 [95% CI: 0.12–1.00]), lower SCIM score (aHR 1st vs 3rd tertile 6.28 [95% CI: 2.03–19.43]), complete SCI (aHR vs incomplete 3.13 [95% CI: 1.05–9.36]) and cancer diagnosis (aHR 2.75 [95% CI: 1.35–5.59]) were further factors associated with non-adherence due to death (Table III, Table SII).

**Table III:** Risk factors for non-adherence due to death

Risk factor	Univariable hazard ratio (95% CI)	*p*-value	Adjusted hazard ratio (95% CI)	*p*-value
Age at spinal cord injury		< 0.0001		0.034
18–30 years	3.08 (0.31–30.37)		2.06 (0.21–19.75)	
31–45 years	Reference		Reference	
46–60 years	6.06 (0.71–51.60)		4.37 (0.40–47.61)	
≥61 years	24.48 (3.21–186.75)		18.47 (1.38–246.39)	
Sex		0.22		0.049
Male	Reference		Reference	
Female	0.63 (0.30–1.32)		0.35 (0.12–1.00)	
Car travel time		> 0.99		0.85
0–1 h	Reference		Reference	
1–2 h	0.97 (0.50–1.87)		1.13 (0.58–2.23)	
2+ h	0.96 (0.39–2.40)		1.32 (0.48–3.68)	
Partnership status on discharge		0.049		0.84
No long-term partner	0.45 (0.21–1.00)		1.09 (0.46–2.59)	
Long-term partner	Reference		Reference	
Spinal Cord Independence Measure III score		< 0.0001		< 0.01
Tertile 1 (0–48)	8.15 (2.93–22.68)		6.28 (2.03–19.43)	
Tertile 2 (49–76)	2.11 (0.66–6.74)		1.95 (0.69–5.51)	
Tertile 3 (77–100)	Reference		Reference	
Lesion aetiology		< 0.01		0.32
Non-traumatic spinal cord injury	2.34 (1.25–4.36)		1.58 (0.64–3.89)	
Traumatic spinal cord injury	Reference		Reference	
Neurological level		0.07		0.22
Cervical	1.75 (0.95–3.24)		0.56 (0.22–1.42)	
Thoracic-sacral	Reference		Reference	
Completeness		0.68		0.041
Complete (AIS A)	0.86 (0.43–1.75)		3.13 (1.05–9.36)	
Incomplete (AIS B–E)	Reference		Reference	
Comorbidity, cancer		< 0.0001		< 0.01
No	Reference		Reference	
Yes	4.77 (2.62–8.68)		2.75 (1.35–5.59)	
Comorbidity, cardiological		0.016		0.58
No	Reference		Reference	
Yes	2.87 (1.22–6.77)		0.73 (0.24–2.24)	
Comorbidity, mental		0.22		0.15
No	Reference		Reference	
Yes	1.47 (0.79–2.71)		1.66 (0.83–3.31)	
Comorbidity, neurological		0.15		0.87
No	Reference		Reference	
Yes	1.74 (0.81–3.72)		1.07 (0.44–2.60)	
Comorbidity, pulmonary		< 0.01		0.35
No	Reference		Reference	
Yes	3.02 (1.47–6.18)		1.48 (0.66–3.33)	

Unadjusted and adjusted hazard ratios from cause-specific Cox regression with general non-adherence (loss to outpatient follow-up) treated as non-informative censoring. Event times are set as the last time that the patient visited the outpatient clinic.

**Fig. 5:**
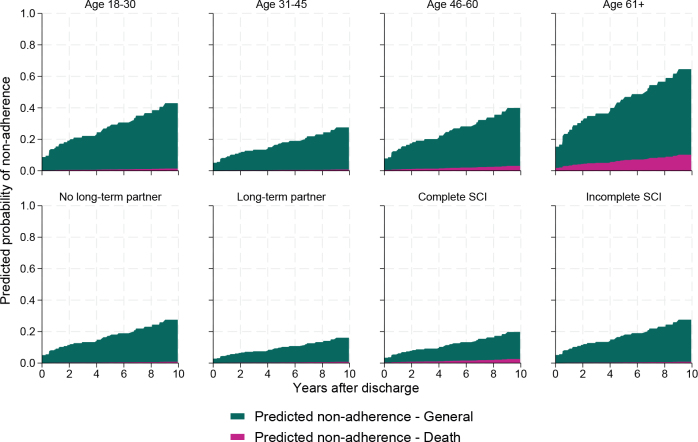
Estimated cumulative incidence function for non-adherence (for general reasons or due to death) according to age group, partnership status, completeness of spinal cord injury (SCI), and presence of cardiological and pulmonary comorbidities. Estimates are based on cause-specific adjusted Cox models, and each graph shows a simulation-based prediction for the respective group variable holding all other covariates constant.

Competing risk regression analysis confirmed that risk factors identified in the cause-specific non-adherence analysis also appear to be associated with the probability (cumulative incidence) of non-adherence during the study period (Table II, Fig. 5). Subhazard ratios from this analysis indicated that cardiological comorbidity, pulmonary comorbidity, and partnership all have a similar association with cumulative incidence, while age over 61 (compared with age 31–45) is estimated to have a larger effect on the cumulative incidence (albeit with less certainty, age global *p* = 0.12).

## DISCUSSION

The present study aimed to investigate the prevalence and time pattern of non-adherence to follow-up care in persons with SCI and explore its associated risk factors. We explored the percentage and timing of non-adherence to follow-up care and a wide range of determinants (including but not limited to injury characteristics, sociodemographic factors, support networks, and geographical limitations) contributing to non-adherence over a period of 10 years after primary inpatient rehabilitation. The prevalence of non-adherence to follow-up care in persons with SCI was substantial, raising concerns about the effectiveness of healthcare management. A considerable proportion of individuals with SCI failed to attend scheduled follow-up visits, leading to gaps in care and suboptimal health and rehabilitation outcomes. We found that, in particular, persons of older age (61 years and older), with longer vehicle travel time to the clinic (over 2 h), or without partners are at risk o non-adherence. Co-morbidities had varying relationships with non-adherence: cardiovascular comorbidities were linked to higher adherence, while pulmonary disease was associated with lower adherence. Death was the main explanation for approximately one-third of the non-adherent cases. Risk of death was especially high in the period shortly after discharge, and among persons in the oldest age group, males, persons with complete SCI, and functional independence in the lowest tertile. When death was incorporated into the analysis as a competing risk, the risk factors for non-adherence did not substantially change, but uncertainty around the contribution of age to the risk of non-adherence increased. Concerning is the finding that in the youngest age group (18–30 years) the predicted non-adherence is 40% after 10 years. Our findings highlight the complex interplay of factors that contribute to attrition rates in this population and underscore the significance of addressing these determinants to enhance patient engagement and continuity of care. This provides a valuable resource for clinicians, researchers, and policymakers to develop evidence-based strategies for enhancing continuity of care and optimizing health and rehabilitation outcomes in this vulnerable population.

These results align with previous research in chronic diseases and SCI specifically, indicating that loss to follow-up care is a persistent issue that warrants urgent attention (23–25). These findings highlight the importance of addressing the challenges associated with follow-up care for individuals with SCI to ensure optimal long-term outcomes.

One of the prominent determinants associated with non-adherence to follow-up care was age. Our study demonstrated that older individuals with SCI were more prone to attrition, as compared with their younger counterparts. This could be due to factors such as need for assistance with travel and during follow-up care and long appointments. Healthcare providers should, therefore, implement tailored strategies to address older patients’ unique challenges, promoting better follow-up adherence. Another concern is adherence in the youngest age group where we found that after 10 years 40% are predicted to be non-adherent to annual follow-up appointments. This number is especially concerning as these individuals have a life with SCI ahead of them and prevention of SHCs is of importance, but also regular follow-up of medication, aids (wheelchair, orthosis), and participation is part of recommended follow-up care. Understanding the healthcare needs in different age groups therefore should be not only a research topic, but also a clinical concern to assure better adherence to follow-up care.

Travel distance (> 2 h by car) emerged as another relevant determinant influencing the likelihood of loss to follow-up care in persons with SCI. Individuals residing in remote or underserved areas faced greater obstacles in accessing healthcare facilities, leading to increased attrition rates. Regional outpatient facilities, telemedicine, and nursing services hold promise in bridging this geographical gap and providing essential medical support to those with limited access to specialized care. Integrating such solutions may significantly contribute to reducing attrition rates and ensuring equitable healthcare delivery.

The presence of comorbidities was found to be related to follow-up care adherence in some cases. Whereas patients with cardiovascular disease were shown to be more adherent to follow-up care, those with pulmonary disease were shown to be less adherent, and cancer, mental, and neurological comorbidities did seem to have a strong relationship to non-adherence. We do not have a specific explanation for this, but it could be at least partially because we offer cardiovascular follow-up care in our clinic, and specific care for chronic pulmonary disease might more often be organized in specialized pulmonary practices.

### Implications for clinicians, research, and policy

Crafting a comprehensive SCI care strategy necessitates a multifaceted approach. The development of the clinical practice guideline for follow-up care (1) was an important step in defining content, setting, and frequency of follow-up care in persons with SCI. The guideline is publicly available on the website of the Association of the Scientific Medical Societies in Germany (AWMF), informing not only SCI specialists, but also general practitioners and persons with SCI. Although implementation of the guideline might not be possible in all healthcare settings (due to financial issues, availability, and accessibility), it might serve as a reference for follow-up care of persons with SCI across the lifespan.

A fundamental step involves understanding the unique needs and behaviours of our diverse clientele. In the telephone interviews we found that various reasons for non-adherence exist, for example due to a bad health condition, and transport problems. In some cases (especially those with AIS D) persons did not feel the need for annual check-ups. By tailoring our initiatives to the specific requirements of individuals with SCI, such as E-appointments, home visits by nurses specialized in SCI, and building robust networks with general practitioners, we can improve adherence to follow-up care. By adopting this collaborative model, healthcare providers empower individuals with SCI, dismantling barriers that often impede consistent follow-up care.

Researchers working within the SCI outpatient context need to be aware that large proportions of the population could be non-adherent to routine clinical care, carefully evaluate whether biases could arise from such non-adherence, and consider how to minimize these biases in the study design phase.

### Key results

Our study highlights the alarming prevalence of non-adherence to follow-up care in persons with SCI even in a high-income country with almost unlimited accessibility to specialized healthcare, and emphasizes the significance of addressing its determinants to enhance patient engagement and healthcare outcomes. Age, travel distance, and partnership status emerged as critical factors contributing to non-adherence rates, necessitating tailored interventions that target each determinant specifically. To combat loss to follow-up care, healthcare providers must adopt a holistic approach that acknowledges the unique challenges faced by individuals with SCI. Integrating telemedicine solutions, enhancing patient education, and involving support networks can play pivotal roles in mitigating the impact of age and travel distance on healthcare adherence. By prioritizing continuity of care and fostering patient empowerment, we can strive towards a more equitable and effective healthcare system that meets the needs of this vulnerable population.

### Limitations

Due to the retrospective design of this study, possible effects of inter- and intrarater variabilities and changed regulations/guidelines over time may have influenced factors that we could not evaluate or control for. Risk factors reflect status on discharge and were not time-updated, meaning that granularity was potentially lost, and change trajectories could not be investigated; nevertheless, proportional hazards testing indicated that this uncollected information did not lead to major violations of modelling assumptions.

Furthermore, newly acquired comorbidities over time are therefore not investigated. Exact dates of death were not available in all cases, so the use of a final rehabilitation visit could have led to some loss of precision in the analyses where death was included, and the role of risk factors that have a strong influence on death in the period shortly after discharge could potentially be underestimated.

The restrictions to accessibility to healthcare due to COVID-19 might have influenced non-adherence. However, the Swiss Paraplegic Centre was closed for planned annual check-ups for only 3 months, and all planned appointments were postponed.

### Generalizability

We do not expect to experience significant under- or oversampling of the population of patients living with SCI in Switzerland, due to the unique role of the study centre in rehabilitation after spinal cord injury in Switzerland. However, because of referral patterns our clinic tends to see a relatively high proportion of the more severe SCI cases in Switzerland, so it is possible that the force of mortality is stronger in this population than in that of other centres. The transferability of the results to clinics and countries with alternative populations, healthcare systems, and different approaches in rehabilitation and follow-up care after spinal cord injury is unclear. Finally, as much of the attrition took place in the first 2 years after discharge, between 2010 and 2014, further investigation focusing on the period soon after discharge is needed to confirm that the risk factors identified here remain relevant in more recent patient cohorts.

## Supplementary Material

NON-ADHERENCE TO FOLLOW-UP CARE IN PERSONS WITH SPINAL CORD INJURY WITHIN 10 YEARS AFTER INITIAL REHABILITATION
