# Supplementary material for: NON-ADHERENCE TO FOLLOW-UP CARE IN PERSONS WITH SPINAL CORD INJURY WITHIN 10 YEARS AFTER INITIAL REHABILITATION
Source: J Rehabil Med. 2024 Aug 26;56:41083. doi: 10.2340/jrm.v56.41083 (PMC11367676; doi:10.2340/jrm.v56.41083)
Supplement: NON-ADHERENCE TO FOLLOW-UP CARE IN PERSONS WITH SPINAL CORD INJURY WITHIN 10 YEARS AFTER INITIAL REHABILITATION [file JRM-56-41083-s1.pdf]

Supplementary material has been published as submitted. It has not been copyedited, or typeset by Journal of Rehabilitation Medicine

Table SI. Risk factors for non-adherence to outpatient follow-up (complete case analysis, N=213)

**Risk factor**	**Univariable hazard ratio (95% CI)**	***p***	**Adjusted hazard ratio (95% CI)**	***p***	**Univariable subhazard ratio (95% CI)**	***p***	**Adjusted subhazard ratio (95% CI)**	***p***
**Age at SCI**		0.07		0.055		0.16		0.10
18-30	1.91 (1.04–3.49)		1.82 (0.97–3.44)		1.80 (1.00–3.25)		1.66 (0.89–3.10)	
31-45	REF		REF		REF		REF	
46-60	1.16 (0.59–2.28)		1.65 (0.74–3.66)		1.06 (0.55–2.07)		1.64 (0.74–3.63)	
61+	1.85 (1.02–3.33)		2.82 (1.25–6.35)		1.30 (0.73–2.34)		2.56 (1.17–5.58)	
**Sex**		0.09		0.25		0.06		0.18
Male	REF		REF		REF		REF	
Female	1.42 (0.95–2.12)		1.30 (0.83–2.05)		1.46 (0.98–2.19)		1.37 (0.87–2.16)	
**Car travel time**		0.11		0.28		0.14		0.36
0-1 hours	REF		REF		REF		REF	
1-2 hours	0.95 (0.61–1.48)		0.89 (0.55–1.44)		0.96 (0.62–1.50)		0.87 (0.54–1.41)	
2+ hours	1.61 (0.97–2.68)		1.48 (0.84–2.61)		1.57 (0.94–2.63)		1.40 (0.79–2.48)	
**Marital status–discharge**		0.039		0.030		0.018		0.020
Not married	1.53 (1.02–2.29)		1.81 (1.06–3.08)		1.63 (1.09–2.44)		1.91 (1.11–3.29)	
Married	REF		REF		REF		REF	
**SCIM III score**		0.60		0.63		0.12		0.22
Tertile 1 (0-48)	0.84 (0.52–1.36)		0.71 (0.35–1.44)		0.60 (0.37–0.98)		0.55 (0.28–1.08)	

Tertile 2 (49-76)	0.80 (0.51–1.27)		0.86 (0.50–1.46)		0.75 (0.48–1.18)		0.82 (0.48–1.40)	
Tertile 3 (77-100)	REF		REF		REF		REF	
**SCI etiology**		0.28		0.75		0.64		>0.99
NTSCI	1.29 (0.81–2.05)		1.11 (0.60–2.05)		1.12 (0.70–1.78)		1.00 (0.55–1.84)	
TSCI	REF		REF		REF		REF	
**Neurological level**		0.22		0.90		0.12		0.67
Cervical	0.78 (0.52–1.16)		1.04 (0.58–1.84)		0.72 (0.48–1.08)		1.13 (0.65–1.96)	
Thoracic-sacral	REF		REF		REF		REF	
**Completeness**		0.19		0.24		0.11		0.15
Complete (AIS A)	1.41 (0.85–2.33)		1.47 (0.77–2.80)		1.51 (0.91–2.51)		1.61 (0.84–3.10)	
Incomplete (AIS B-E)	REF		REF		REF		REF	
**Comorbidity – cancer**		0.45		0.54		0.10		0.22
No	REF		REF		REF		REF	
Yes	0.76 (0.37–1.55)		0.77 (0.33–1.79)		0.54 (0.26–1.13)		0.59 (0.26–1.36)	
**Comorbidity – cardiological**		0.023		<0.01		<0.01		0.015
No	REF		REF		REF		REF	
Yes	0.64 (0.43–0.94)		0.52 (0.33–0.84)		0.57 (0.38–0.84)		0.55 (0.34–0.89)	
**Comorbidity – mental**		0.96		0.85		0.76		0.82
No	REF		REF		REF		REF	
Yes	1.01 (0.66–1.54)		1.05 (0.64–1.73)		0.94 (0.61–1.43)		0.94 (0.57–1.56)	
**Comorbidity–neurological**		0.22		0.92		0.09		0.88

No	REF	REF	REF	REF
Yes	0.78 (0.52–1.17)	1.03 (0.63–1.66)	0.71 (0.47–1.06)	0.96 (0.59–1.56)
**Comorbidity–pulmonary**	0.32	0.024	0.79	0.026
No	REF	REF	REF	REF
Yes	1.22 (0.82–1.81)	1.75 (1.08–2.84)	1.06 (0.71–1.56)	1.72 (1.07–2.78)

Unadjusted and adjusted hazard ratios from cause-specific Cox regression with death treated as non-informative censoring, as well as unadjusted and adjusted sub-hazard ratios from a competing risk regression treating death as a competing event. All listed covariates were included in the adjusted analyses.

NTSCI: non-traumatic spinal cord injury; SCI: spinal cord injury; TSCI: traumatic spinal cord injury.

Table SII. Risk factors for non-adherence due to death (complete case analysis, n=213)

**Risk factor**	**Univariable hazard ratio (95% CI)**	***p***	**Adjusted hazard ratio (95% CI)**	***p***
**Age at SCI**		<0.0001		0.14
18-30	3.08 (0.31–30.37)		2.56 (0.28–23.02)	
31-45	REF		REF	
46-60	6.06 (0.71–51.60)		4.11 (0.39–42.77)	
61+	24.48 (3.21–186.75)		13.07 (0.96–178.89)	
**Sex**		0.22		0.11
Male	REF		REF	
Female	0.63 (0.30–1.32)		0.39 (0.12–1.23)	
**Car Travel Time**		>0.99		0.98
0-1 hours	REF		REF	
1-2 hours	0.97 (0.50–1.87)		1.05 (0.51–2.19)	
2+ hours	0.96 (0.39–2.40)		1.11 (0.30–4.15)	
**Marital status – discharge**		0.056		0.91
Not married	0.46 (0.21–1.02)		1.05 (0.45–2.45)	
Married	REF		REF	
**SCIM Score**		<0.0001		<0.01
Tertile 1 (0-48)	8.15 (2.93–22.68)		6.60 (2.00–21.79)	
Tertile 2 (49-76)	2.11 (0.66–6.74)		2.18 (0.77–6.22)	
Tertile 3 (77-100)	REF		REF	
**SCI etiology**		<0.01		0.22
NTSCI	2.34 (1.25–4.36)		1.87 (0.68–5.09)	
TSCI	REF		REF	
**Neurological level**		0.07		0.20
Cervical	1.75 (0.95–3.24)		0.52 (0.19–1.42)	
Thoracic-sacral	REF		REF	
**Completeness**		0.68		0.25
Complete (AIS A)	0.86 (0.43–1.75)		0.49 (0.15–1.64)	
Incomplete (AIS B-E)	REF		REF	
**Comorbidity – cancer**		<0.0001		<0.01
No	REF		REF	

Yes	4.77 (2.62–8.68)		3.39 (1.47–7.84)	
**Comorbidity–cardiological**		0.016		0.47
No	REF		REF	
Yes	2.87 (1.22–6.77)		0.66 (0.22–2.01)	
**Comorbidity – mental**		0.22		0.22
No	REF		REF	
Yes	1.47 (0.79–2.71)		1.61 (0.75–3.47)	
**Comorbidity–neurological**		0.15		0.77
No	REF		REF	
Yes	1.74 (0.81–3.72)		1.15 (0.44–3.03)	
**Comorbidity–pulmonary**		<0.01		0.57
No	REF		REF	
Yes	3.02 (1.47–6.18)		1.30 (0.53–3.20)	

Unadjusted and adjusted hazard ratios from cause-specific Cox regression with general non-adherence (loss to outpatient follow-up) treated as non-informative censoring. Event times are set as the last time that the patient visited the outpatient clinic. All listed covariates were included in the adjusted analysis.

NTSCI: non-traumatic spinal cord injury; SCI: spinal cord injury; TSCI: traumatic spinal cord injury.
